# The effect of different dietary ratios of lysine and arginine in diets with high or low methionine levels on oxidative and epigenetic DNA damage, the gene expression of tight junction proteins and selected metabolic parameters in *Clostridium perfringens*-challenged turkeys

**DOI:** 10.1186/s13567-020-00776-y

**Published:** 2020-04-07

**Authors:** Katarzyna Ognik, Paweł Konieczka, Dariusz Mikulski, Jan Jankowski

**Affiliations:** 1 Department of Biochemistry and Toxicology, University of Life Sciences, 20-950 Lublin, Poland; 2 Department of Poultry Science, University of Warmia and Mazury in Olsztyn, 10-719, Olsztyn, Poland

## Abstract

Two experiments were performed to investigate the effect of different ratios of arginine (Arg) to lysine (Lys) in diets with low (30% Lys; Experiment 1) and high (45% Lys; Experiment 2) methionine (Met) levels on selected metabolic parameters, oxidative and epigenetic DNA damage, and the mechanisms underlying intestinal barrier integrity in turkeys challenged with *Clostridium perfringens*. In each experiment, 108 one-day-old Hybrid Converter female turkeys were placed in 6 pens (18 birds per pen) and reared for 42 days. At 34, 36 and 37 days of age, half of the birds were subjected to *C. perfringens* challenge. A 3 × 2 factorial design with three levels of Arg relative to Lys (90, 100 and 110%; Arg_90_, Arg_100_ and Arg_110_, respectively) and *C. perfringens* infection (−, +) was employed. Challenging birds with *C. perfringens* increased lipid oxidation and the oxidation and methylation of DNA of intestinal mucosa, and down-regulated the activities of DNA-repairing enzymes. Neither the dietary treatment nor the challenge affected the markers of liver function or metabolism. Arg_110_ diets with the high Met level induced DNA oxidation and methylation whereas these processes were downregulated in birds fed Arg_90_ diets. The results indicate that Arg_90_ diets with high Met levels have a beneficial influence on the indicators of intestinal barrier integrity in turkeys with necrotic enteritis (NE). Despite the analyzed amino acid ratios interacted with the systems responsible for the maintenance of gut integrity in the host organism, this dietary intervention probably enabled birds to cope with NE.

## Introduction

In turkeys, necrotic enteritis (NE) is caused by *Clostridium perfringens* (*C. perfringens*) anaerobic bacteria and their toxins, and it causes considerable economic losses in poultry farming [1]. In a post-mortem analysis, the disease manifests by strong inflammation and necrosis of the intestinal mucosa, which impairs nutrient absorption, weakens immune and antioxidant systems and, consequently, compromises growth performance and increases bird mortality [2–4]. Pathological changes associated with NE are also observed in parenchymal organs such as the liver, which can disrupt many biochemical processes. NE can be effectively prevented with organic acids [5] or upon treatment with antibiotics [6].

After the introduction of a ban on the use of antibiotics as growth promoters, poultry nutrition became a viable option for preventing NE. According to many authors, diet has a considerable influence (around 57% relative to the impact of genetic factors which is estimated at 12%) on the gut microbial community [7]. Research has demonstrated that reduced intake or elimination of non-starch polysaccharides (NSP), i.e. complex carbohydrates that increase the viscosity of intestinal digesta, from the diet can intensify fermentation processes and the proliferation of bacteria, including *C. perfringens* [8]. Bacterial and viral infections exert immunosuppressive effects in poultry [9, 10] and induce reactions that lead to oxidative stress in cells [11, 12]. According to recent research, the supplementation of poultry diets with the appropriate amounts of amino acids, such as arginine (Arg) and methionine (Met), can promote intestinal barrier integrity in many infectious diseases [13–16] because these amino acids stimulate immune and antioxidant systems [17–21]. Tan et al. [14, 22] found that diets supplemented with Arg minimize the damage to intestinal villi and crypts caused by coccidiosis in broiler chickens. Zhang et al. [3] demonstrated that higher levels of l-arginine in the diet improved intestinal barrier integrity and modulated gut microflora in chickens challenged with *C. perfringens*, producing a consortium that was like to that of healthy controls with higher counts of beneficial bacteria and reduced counts of harmful species. According to Zhang et al. [16], Arg protects the intestinal mucosa by stimulating non-specific immunity, improving intestinal absorption and inhibiting *C. perfringens* colonization in broilers with inflammatory bowel disease. Munir et al. [9] also demonstrated that Arg could act as an immunoregulator against hydropericardium syndrome virus (HPSV) as well as other poultry pathogens. Our previous study of turkeys [12] revealed that dietary supplementation with Met at a concentration approximately 50% higher than that recommended by the NRC [23] intensified oxidative processes in the intestines, but also stimulated antioxidant mechanisms in the blood and livers of turkeys with HE. However, Ruth and Field [13] argued that dietary supplementation with Met in excess of the recommended levels [23] decreases symptoms of oxidative stress in the intestines of birds with viral and bacterial infections. The cited research is inconclusive and merits further investigation on whether dietary supplementation with the appropriate proportions of Arg and Met can limit the symptoms of oxidative stress, minimize epigenetic alterations that cause DNA damage in turkeys infected with *C. perfringens* and, consequently, improve intestinal barrier integrity.

According to the NRC [23], the inclusion rate of Arg in turkey diets should reach 90–100% of Lys content, whereas turkey breeding companies [24] recommend higher Arg inclusion rates at 102–105% of Lys content. The Met inclusion rate has been set at 30–38% of Lys content by the NRC [23] and at 36–41% by turkey breeders. Although intestinal barrier and intestinal permeability are important for health and disease, the mucosal barrier and its role in enteric disease are still poorly understood in turkeys. Therefore, the molecular basis of differential responses to infections caused by certain microorganisms in the turkey gut should be elucidated because they are critical for the birds’ health. To the best of our knowledge, the dietary inclusion levels of limiting amino acids (Lys, Arg and Met) and their optimal ratios in the diets of young turkeys challenged with *C. perfringens* have not been reported in the literature to date. We hypothesized that the optimal concentrations and ratios of Lys, Arg and Met in turkey diets can limit oxidation and methylation of biologically important molecules.

The aim of this study was to determine the effect of different ratios of arginine (Arg) to lysine (Lys) in diets with low (30% Lys; Experiment 1) and high (45% Lys; Experiment 2) methionine (Met) levels on selected metabolic parameters, oxidative and epigenetic DNA damage, and the mechanisms underlying intestinal barrier integrity in turkeys challenged with *C. perfringens.*

## Materials and methods

The study protocol was approved by the Local Ethics Committee (University of Warmia and Mazury, Olsztyn, Poland), and the animals were cared for under guidelines comparable to those laid down by the EU Directive 2010/63/EU.

### Birds and housing

A total of 216 one-day-old Hybrid Converter female turkey poults obtained from a commercial hatchery (Grelavi company in Ketrzyn, NE, Poland) were randomly placed in 12 pens on litter (4 m^2^ each; 2.0 m × 2.0 m), and were reared to 42 days of age. The initial BW of 1-day-old poults was 55.7 ± 0.1 g. The birds were divided into 2 subgroups (referred thereafter as Experiment 1 and Experiment 2), each subgroup consisted of 6 pens with 18 birds per pen. At 34, 36 and 37 days of age, half of the birds were subjected to *C. perfringens* challenge. The temperature and lighting programs were consistent with the recommendations for standard management practices [24].

### Experimental design and diets

During the 6-week experiment, birds were fed ad libitum isocaloric diets that met or exceeded their requirements, containing high levels of Lys, approximately 1.80% and 1.65% in two successive feeding periods, consistent with the Lys levels recommended in the Management Guidelines for Raising Commercial Turkeys [24]. The experiments had a completely randomized 3 × 2 factorial design with three levels of Arg (90%, 100% and 110% relative to the content of dietary Lys; Arg_90_, Arg_100_ and Arg_110_, respectively) and *C. perfringens* infection (−, +). The diets in Experiment 1 had low Met content (30% Lys), and the diets in Experiment 2 had high Met content (45% of Lys). The AA content of basal diets was determined, and they were mixed with adequate amounts of the above AA. The Lys, Met and Arg content of experimental diets was determined analytically again (Table 1). The diets were offered as crumbles (days 1–28) and pellets (days 29–42).

**Table 1:** **Analyzed lysine (Lys), arginine (Arg) and methionine (Met) content of turkey diets, g/100** **g.**

Treatment	Amino acid	Experiments 1 and 2	
		Week 1–4	Week 5–6
Arg_90_	Lys	1.81	1.67
	Arg	1.59	1.50
	Met	0.53	0.50
Arg_100_	Lys	1.85	1.64
	Arg	1.86	1.64
	Met	0.56	0.52
Arg_110_	Lys	1.89	1.65
	Arg	2.04	1.77
	Met	0.57	0.51

### *Clostridium perfringens* challenge

At 34, 36 and 37 days of age, birds were infected with 1 mL (*per os* directly into the crop) of *C. perfringens* type A strain 56 containing approximately 10^8^ CFU/mL cultured in a brain heart infusion medium (Sigma Aldrich) whereas the non-challenged group received the same dose of sterilized broth medium. The *C. perfringens* challenge was preceded by administering 1 mL of a coccidia vaccine containing Eimeria (E) species: *E. acervulina*, 5000 oocytes; *E. maxima*, 3500 oocytes; *E. mitis*, 5000 oocytes; *E. praecox*, 5000 oocytes; *E. tenella*, 5000 oocytes (Laboratorios HIPRA S.A., Spain) to turkeys at 31 and 34 days of age to make favorable environment in the gut for *C. perfringens* colonization.

### Sample collection and laboratory analysis

At 42 days of age, birds were weighted individually, and 8 turkeys from each treatment were sacrificed by cervical dislocation, and the abdominal cavity was opened for ileal (middle-ileum) tissue collection and processing. Blood samples were taken from 8 birds per group. Blood samples were collected into test tubes with an anticoagulant (heparin) from the wing vein, they were centrifuged at 3000 × *g* for 10 min, and blood plasma was collected for further analysis.

### RNA extraction and real-time quantitative PCR

RNA isolation was performed in accordance with previously developed procedures with some modifications [25]. Briefly; total RNA from the ilium was isolated using the GeneMATRIX Universal RNA Purification Kit (EURX Ltd., Gdańsk, Poland) according to the manufacturer’s protocol. The isolated RNA yield was estimated spectrophotometrically (Nanodrop, NanoDrop Technologies, Wilmington, DE, USA), with integrity assessed electrophoretically by separation on agarose gel. For complementary cDNA synthesis, 800 ng/mL of RNA from ileal tissue was reverse transcribed using the NG dART RT kit (EURX Ltd., Gdańsk, Poland) according to the manufacturer’s instructions. Specific primers for respective genes were designed using Primer 3 software (Whitehead Institute, Cambridge, MA, USA) and synthesized by Genomed (Warsaw, Poland). The respective primer sequences are shown in Table 2.

**Table 2:** **Genes and primers used in the study.**

Gene	Primer	Sequence (5′-3′)	Melting temperature (°C)	Product size (nt)	GenBank access no.
ACTB	Forward	TACCCCATTGAACACGGCAT	58	96	NM_001303173
	Reverse	CTCCTCAGGGGCTACTCTCA			
GAPDH	Forward	AGGATACACAGAGGACCAGGTTG	58	71	NM_001303179
	Reverse	CCGCATCAAAGGTGGAGGAATG			
ZO-1	Forward	AGAGGCAACTGAACCATAG	58	114	XM_019619275
	Reverse	CTGCTGAGAGGCTAATACAA			
GLP2	Forward	GCAGTGAAGGAGAAGTGA	58	200	XM_010721176
	Reverse	GAGGCTGTAAGAAGTAGGA			
OCLN	Forward	GCAGATGTCCAGCAGTTA	55	127	XM_019610822
	Reverse	GTTCACACTCACCTCCTG			
TFF2	Forward	AAAATAGCAGCCAGGGAGCG	58	92	XM_010724393
	Reverse	ACTGACGCATTGAAGCAGCA			
CLDN15	Forward	GCAAGGAGGCTTCTGAAA	55	161	XM_019618905
	Reverse	CAGTAACTATGTGGCAAGGT			
OGG1	Forward	GGGACAAATGGGCACCTG	58	102	XM_010718590
	Reverse	GCAGAGGCAATAGGCTCAG			

ACTB: β-actin, GAPDH: Glyceraldehyde-3-Phosphate Dehydrogenase, ZO-1: Zonula occludens-1, GLP2: Glucagon-like peptide-2, OCLN: Occludin, TFF2: Trefoil Factor 2, CLDN15: Claudin 15, and OGG1: 8-Oxoguanine glycosylase.

Real-time qPCR was performed using the BioRad CFX 96 thermocycler (Bio-Rad Laboratories, Inc., CA, USA) according to the following protocol: one cycle at 95 °C for 15 min (enzyme activation), followed by a PCR including 35 cycles at 95 °C for 10 s (denaturation), 55–58 °C for 10 s (annealing), and 72 °C for 20 s (elongation). A melting curve analysis was performed over 65–95 °C at 0.1 °C/s intervals. Negative controls without the cDNA template were included in each reaction. Real-time qPCR for each cDNA sample was performed in duplicate. Normalized gene expression was calculated using the comparative quantitation option of Rotor qPCR Biorad CFX 96 (Bio-Rad Laboratories, Inc., CA, USA), and determined using the supplied Expression Software Tool. The glyceraldehyde-3-phosphate dehydrogenase (GAPDH) and β-actin (ACTB) genes were selected as endogenous control genes to normalize gene expression.

### DNA oxidation and methylation, and metabolic parameters

The levels of 8-hydroxydeoxyguanosine (8-OHdG), endonuclease 1 (APE-1), and glycosylases TDG and ANPG were determined in the wall of the ileum and in the blood of turkeys using OxiSelect diagnostic kits (Cell Biolabs, Inc., San Diego, USA). DNA was isolated from the blood and intestinal wall using QIAGEN kits. Epigenetic changes in the blood and intestinal wall were determined by analyzing global DNA methylation (methylome) using Sigma Aldrich diagnostic kits. The concentration of malondialdehyde (MDA) in the blood of turkeys was determined using kits produced by Cell Biolabs, Inc. (San Diego, USA). The activity of superoxide dismutase (SOD) and glutathione peroxidase (GPx) in the blood of turkeys was determined by spectrometry using Ransel and Ransod diagnostic kits manufactured by Randox (Poland). The plasma concentrations of total cholesterol (TC), triglycerides (TG), uric acid (UA), urea (UREA), total protein (TP), albumin (ALB) and glucose (GLU), and the activity of alanine aminotransferase (ALT), aspartate aminotransferase (AST) and creatine kinase (CK) were measured using an automated biochemistry analyzer (Plasma Diagnostic Instruments Horiba, Kyoto, Japan).

### Statistical analysis

Both experiments were performed in a completely randomized 3 × 2 factorial design, and the data were subjected to 2-way ANOVA to examine the following effects: (a) main effect of three levels of Arg (Arg_90_, Arg_100_ and Arg_110_); (b) main effect of *C. perfringens* infection (−, +); and (c) interaction between Arg inclusion levels and *C. perfringens* infection. All data were analyzed using the GLM procedure of STATISTICA software version 12. When a significant interaction effect was noted, Tukey’s test was used to determine differences between the experimental factors. Data variability was expressed as pooled standard errors of the mean (SEM), and *P* < 0.05 was considered statistically significant.

## Results

### Experiment 1

#### Effects of infection

*Clostridium perfringens* infection did not decrease the BW of turkeys at 6 weeks of age (2.449 vs. 2.367 kg, *P* = 0.167). The expression of occludin, ZO-1, GLP2, OGG1 and TFF2 genes increased in the wall of the ileum (*P* < 0.001) in response to *C. perfringens* infection (Table 3). In comparison with uninfected turkeys, *C. perfringens* infection increased 8-OHdG levels and decreased the activity of APE-1, TDG and ANPG in the wall of the ileum (*P* < 0.001). Higher levels of 8-OHdG, a higher percentage of methylated DNA (*P* < 0.001, respectively) and lower activity of APE-1 (*P* = 0.003) were also noted in the blood of infected turkeys (Table 4). *Clostridium perfringens* infection increased the levels of MDA (*P* = 0.005) and ALB (*P* < 0.001), and GPx activity (*P* < 0.001), and decreased the levels of TG (*P* < 0.001) and UA (*P* < 0.001), and ALT activity (*P* < 0.001) (Table 5).

**Table 3:** **Gene expression in the ileal tissues of 42-day-old turkeys fed diets with different arginine (Arg) to lysine (Lys) ratios and a low methionine (Met) level, challenged with*Clostridium perfringens*—(Experiment 1).**

Item	ZO-1	Occludin	GLP2	OGG1	TFF2
Arg_90_*Infection (−)	0.655	0.005	0.331^b^	0.325	0.003
Arg_90_*Infection (+)	2.191	0.013	1.500^a^	0.711	0.009
Arg_100_*Infection (−)	0.762	0.003	0.429^ab^	0.142	0.004
Arg_100_*Infection (+)	1.892	0.014	0.933^ab^	0.569	0.010
Arg_110_*Infection (−)	0.933	0.007	0.712^ab^	0.211	0.004
Arg_110_*Infection (+)	2.434	0.025	1.001^a^	1.197	0.011
SEM	0.124	0.002	0.080	0.074	0.0007
Arg level, %					
90	1.423	0.009	0.915	0.518^ab^	0.006
100	1.327	0.008	0.681	0.355^b^	0.007
110	1.684	0.016	0.856	0.704^a^	0.008
*C. perfringens infection*^1^					
–	0.783^b^	0.005^b^	0.490^b^	0.226^b^	0.003^b^
+	2.172^a^	0.017^a^	1.145^a^	0.826^a^	0.010^a^
*P* value					
Arg	0.108	0.052	0.252	0.045	0.420
*Infection*	0.001	0.001	0.001	0.001	0.001
Arg × *infection*	0.425	0.295	0.011	0.057	0.868

Diets contained three levels of Arg relative to Lys (90, 100 and 110%; Arg_90_, Arg_100_ and Arg_110_, respectively) and a low level of Met (30% dietary Lys).

^1^At 34, 36 and 37 days of age, birds were infected with 1 mL (*per os* directly into the crop) of a culture medium of *C. perfringens* type A strain 56 containing approximately 10^8^ CFU/mL of bacteria. ^a,b^Means within a row with different superscripts differ significantly (*P* < 0.05).

**Table 4:** **Oxidative and epigenetic DNA damage and the activities of repair enzymes in the blood and ileal tissues of 42-day-old turkeys fed diets with different arginine (Arg) to lysine (Lys) ratios and a low methionine (Met) level, challenged with*Clostridium perfringens*—(Experiment 1).**

Item	Ileal tissue					Blood				
	8-OHdG pg/µL DNA	Methylation DNA, %	APE-1 ng/g	TDG ng/g	ANPG ng/g	8-OHdG pg/mL	Methylation DNA, %	APE-1 ng/mL	TDG ng/mL	ANPG ng/mL
Arg_90_*Infection (−)	24.26^d^	14.92^c^	1731.0^a^	1056.9	39.01	4.424^c^	28.66	160.1	83.68	4.197
Arg_90_*Infection (+)	53.44^b^	18.72^ab^	933.2^c^	596.3	17.78	6.825^a^	58.23	146.7	83.99	4.361
Arg_100_*Infection (−)	28.04^d^	15.37^bc^	1746.2^a^	994.5	42.41	5.061^bc^	30.14	182.5	100.30	4.747
Arg_100_*Infection (+)	86.21^a^	19.55^a^	995.3^c^	658.9	21.07	6.031^ab^	46.46	152.2	92.31	4.601
Arg_110_*Infection (−)	38.08^c^	19.00^ab^	1382.5^b^	876.3	37.38	5.711^abc^	30.20	147.4	94.87	4.772
Arg_110_*Infection (+)	84.96^a^	13.21^c^	1018.2^c^	651.0	23.16	6.017^ab^	52.06	144.8	93.37	4.978
SEM	3.757	0.493	57.80	33.85	1.673	0.175	2.252	3.025	1.607	0.075
Arg level, %										
90	38.85^b^	16.82	1332.1	826.6	28.39	5.624	43.44	153.4^ab^	83.83^b^	4.279^b^
100	57.12^a^	17.46	1370.7	826.7	31.74	5.546	38.30	167.4^a^	96.31^a^	4.674^ab^
110	59.96^a^	16.11	1200.3	763.6	30.27	5.864	41.13	146.1^b^	94.12^a^	4.875^a^
*C. perfringens infection*^1^										
–	30.13^b^	16.43	1619.9^a^	975.9^a^	39.60^a^	5.065^b^	29.67^b^	163.3^a^	92.95	4.572
+	74.43^a^	17.16	982.2^b^	635.4^b^	20.67^b^	6.291^a^	52.25^a^	147.9^b^	89.89	4.647
*P* value										
Arg	0.001	0.329	0.075	0.433	0.356	0.641	0.399	0.004	0.002	0.003
*Infection*	0.001	0.327	0.001	0.001	0.001	0.001	0.001	0.003	0.292	0.589
Arg × *infection*	0.001	0.001	0.012	0.119	0.222	0.014	0.221	0.084	0.468	0.523

Diets contained three levels of Arg relative to Lys (90, 100 and 110%; Arg_90_, Arg_100_ and Arg_110_, respectively) and a low level of Met (30% dietary Lys).

^1^At 34, 36 and 37 days of age, birds were infected with 1 mL (*per os* directly into the crop) of a culture medium of *C. perfringens* type A strain 56 containing approximately 10^8^ CFU/mL of bacteria. ^a,b,c^Means within a row with different superscripts differ significantly (*P* < 0.05).

**Table 5:** **Metabolic parameters in the blood of 42-day-old turkeys fed diets with different arginine (Arg) to lysine (Lys) ratios and a low methionine (Met) level, challenged with*Clostridium perfringens*—(Experiment 1).**

Item	CK U/L	ALT U/L	AST U/L	SOD U/gHb	GPx U/gHb	TP g/L	ALB g/L	UA mmol/L	UREA mmol/L	TC mmol/L	TG mmol/L	MDA µmol/L	GLU mmol/L
Arg_90_*Infection (−)	0.255	9.15^b^	250.1^c^	1029.4	14.23^bc^	39.61^ab^	16.69^b^	0.424^ab^	1.019	4.069	0.676	1.905^a^	9.45^c^
Arg_90_*Infection (+)	0.233	10.52^b^	261.4^bc^	825.5	17.06^b^	41.02^a^	18.60^a^	0.341^bc^	0.789	3.794	0.461	1.886^ab^	11.59^ab^
Arg_100_*Infection (−)	0.264	12.57^a^	300.0^a^	1024.8	15.17^bc^	28.57^c^	13.50^c^	0.408^ab^	1.171	3.532	0.533	2.014^a^	11.78^a^
Arg_100_*Infection (+)	0.264	12.71^a^	279.1^ab^	745.2	31.91^a^	34.73^b^	18.89^a^	0.371^abc^	0.942	3.406	0.354	2.089^a^	10.02^bc^
Arg_110_*Infection (−)	0.275	14.43^a^	301.1^a^	1082.6	15.30^bc^	38.07^ab^	18.41^a^	0.447^a^	1.299	3.250	0.836	1.395^b^	11.83^a^
Arg_110_*Infection (+)	0.266	6.43^c^	248.3^c^	1364.5	13.07^c^	36.14^ab^	19.51^a^	0.295^c^	1.095	3.410	0.688	2.209^a^	9.80^c^
SEM	0.006	0.468	4.067	57.66	0.999	0.742	0.325	0.011	0.062	0.070	0.025	0.059	0.215
Arg level, %													
90	0.244	9.83^b^	255.8^b^	927.5^b^	15.65^b^	40.32^a^	17.65^b^	0.382	0.904	3.931^a^	0.568^b^	1.896	10.52
100	0.264	12.64^a^	289.5^a^	885.0^c^	23.54^a^	31.65^b^	16.19^c^	0.390	1.057	3.469^b^	0.444^c^	2.051	10.90
110	0.271	10.43^ab^	274.7^ab^	1223.6^a^	14.18^b^	37.10^ab^	18.96^a^	0.371	1.197	3.330^b^	0.762^a^	1.802	10.81
*C. perfringens infection*^1^													
–	0.149	12.05^a^	283.7^a^	1045.6	14.90^b^	35.42	16.20^b^	0.426^a^	1.163	3.617	0.682^a^	1.77^b^	11.02
+	0.254	9.89^b^	262.9^b^	978.4	20.68^a^	37.30	19.00^a^	0.336^b^	0.942	3.537	0.501^b^	2.06^a^	10.47
*P* value													
Arg	0.149	0.001	0.001	0.024	0.001	0.001	0.001	0.638	0.158	0.001	0.001	0.121	0.626
*Infection*	0.372	0.001	0.001	0.528	0.001	0.054	0.001	0.001	0.077	0.518	0.001	0.005	0.104
Arg × *infection*	0.734	0.001	0.001	0.074	0.001	0.005	0.001	0.020	0.995	0.348	0.451	0.002	0.000

Diets contained three levels of Arg relative to Lys (90, 100 and 110%; Arg_90_, Arg_100_ and Arg_110_, respectively) and a low level of Met (30% dietary Lys).

^1^At 34, 36 and 37 days of age, birds were infected with 1 mL (*per os* directly into the crop) of a culture medium of *C. perfringens* type A strain 56 containing approximately 10^8^ CFU/mL of bacteria. ^a,b,c^Means within a row with different superscripts differ significantly (*P* < 0.05).

#### Effects of dietary ratios of Arg to Lys

Different dietary inclusion levels of Arg had no effect on the BW of turkeys at 6 weeks of age (2.383 vs. 2.429 vs. 2.412 kg, *P* = 0.808). Diets with the highest Arg content (110% Lys) increased the expression of the OGG1 gene (*P* = 0.004) in the wall of the ileum. An Arg × infection interaction was noted for the expression of the GLP2 gene in the ileum (*P* = 0.045): *C. perfringens* infection increased the expression of the GLP2 gene at the lowest dietary Arg content (90% Lys), but not at the medium or highest Arg content (100% and 110% Lys, respectively) (Table 3).

In comparison with turkeys fed Arg_90_ diets, diets with higher Arg content increased 8-OHdG levels (*P* < 0.001) in the ileum. The lowest activity of APE-1 (*P* = 0.004) was noted in turkeys fed Arg_110_ diets, and the lowest activity of TDG (*P* = 0.002) and ANPG (*P* = 0.003) was observed in birds receiving Arg_90_ diets. An Arg × infection interaction was noted for DNA methylation in the wall of the ileum (*P* < 0.001): *C. perfringens* infection decreased DNA methylation at the highest dietary Arg content (Arg_110_), but not at the medium or lowest Arg content (Arg_100_ and Arg_90_, respectively). An Arg × infection interaction was also found for 8-OHdG levels in the blood (*P* = 0.014): *C. perfringens* infection increased 8-OHdG levels at the lowest dietary Arg content (Arg_90_), but not at the medium or highest Arg content (Arg_100_ and Arg_110_, respectively) (Table 4).

Diets with the lowest Arg content increased TP levels in the blood of turkeys (*P* < 0.001) and decreased AST activity (*P* < 0.001). In comparison with diets with the lowest and medium Arg inclusion rates, diets with the highest Arg content increased SOD activity (*P* = 0.024). Turkey fed Arg_90_ diets were characterized by the lowest ALT activity and the highest TC concentration (*P* < 0.001). The plasma levels of ALB and TG increased (both *P* < 0.001) with increasing Arg content. An Arg × infection interaction was noted for ALT and AST activity (both *P* < 0.001): *C. perfringens* infection decreased the activity of both enzymes at the highest dietary Arg content (Arg_110_), but not at the medium or lowest Arg content (Arg_100_ and Arg_90_, respectively). An Arg × infection interaction was observed for TP levels and GPx activity (both *P* < 0.001): *C. perfringens* infection increased TP levels and GPx activity at the medium dietary Arg content (Arg_100_), but not at the lowest or highest Arg content (Arg_90_ and Arg_110_, respectively). An Arg × infection interaction was also found for ALB levels in the blood plasma of turkeys (*P* < 0.001): *C. perfringens* infection increased ALB levels at the lowest and medium Arg content (Arg_90_ and Arg_100_, respectively), but not at the highest Arg content (Arg_110_). The following Arg × infection interaction was noted for the plasma levels of UA and MDA (both *P* = 0.002): *C. perfringens* infection increased UA and MDA levels at the highest dietary Arg content (Arg_110_), but not at the lowest or medium Arg content (Arg_90_ and Arg_100_, respectively). The following Arg × infection interaction was observed for GLU levels in the blood plasma of turkeys (*P* < 0.001): *C. perfringens* infection increased GLU levels at the lowest dietary Arg content (Arg_90_), but not at the medium or highest Arg content (Arg_100_ and Arg_110_, respectively) (Table 5).

### Experiment 2

#### Effects of infection

Similarly to Experiment 1, *C. perfringens* infection had no influence on the BW of turkeys (2.467 vs. 2.392 kg, *P* = 0.307). *Clostridium perfringens* infection increased the expression of occludin, ZO-1, GLP2, OGG1 and TFF2 genes (*P* < 0.001) in the wall of the ileum (Table 6). Similarly to Experiment 1, *C. perfringens* infection increased 8-OHdG levels and decreased the activity of APE-1, TDG and ANPG in the wall of the ileum (*P* < 0.001). The infection increased 8-OHdG levels and the percentage of methylated DNA (*P* < 0.001), and decreased APE-1 activity (*P* = 0.01) also in turkeys fed diets containing 45% Met. In comparison with uninfected turkeys, *C. perfringens* infection decreased the activity of ANPG in the blood (*P* = 0.004) and increased the percentage of methylated DNA in the wall of the ileum (*P* = 0.004) (Table 7).

**Table 6:** **Gene expression in the ileal tissues of 42-day-old turkeys fed diets with different arginine (Arg) to lysine (Lys) ratios and a high methionine (Met) level, challenged with*Clostridium perfringens*—(Experiment 2).**

Item	ZO-1	Occludin	GLP2	OGG1	TFF2
Arg_90_*Infection (−)	0.794	0.005	0.375	0.184	0.003
Arg_90_*Infection (+)	1.918	0.022	1.145	0.666	0.011
Arg_100_*Infection (−)	0.841	0.006	0.385	0.172	0.003
Arg_100_*Infection (+)	2.485	0.014	1.590	0.545	0.009
Arg_110_*Infection (−)	0.838	0.005	0.368	0.283	0.003
Arg_110_*Infection (+)	2.253	0.014	1.219	0.616	0.007
SEM	0.120	0.001	0.091	0.044	0.0007
Arg level, %					
90	1.356	0.013	0.760	0.425	0.007
100	1.663	0.010	0.987	0.359	0.006
110	1.545	0.009	0.793	0.450	0.005
*C. perfringens infection*^1^					
–	0.824^b^	0.005^b^	0.376^b^	0.213^b^	0.003^b^
+	2.219^a^	0.017^a^	1.318^a^	0.609^a^	0.009^a^
*P* value					
Arg	0.134	0.144	0.259	0.532	0.421
*infection*	0.001	0.001	0.001	0.001	0.001
Arg × *infection*	0.237	0.078	0.301	0.654	0.283

Diets contained three levels of Arg relative to Lys (90, 100 and 110%; Arg_90_, Arg_100_ and Arg_110_, respectively) and a high level of Met (45% dietary Lys).

^1^At 34, 36 and 37 days of age, birds were infected with 1 mL (*per os* directly into the crop) of a culture medium of *C. perfringens* type A strain 56 containing approximately 10^8^ CFU/mL of bacteria. ^a,b^Means within a row with different superscripts differ significantly (*P* < 0.05).

**Table 7:** **Oxidative and epigenetic DNA damage and the activities of repair enzymes in the blood and ileal tissues of 42-day-old turkeys fed diets with different arginine (Arg) to lysine (Lys) ratios and a high methionine (Met) level, challenged with*Clostridium perfringens*—(Experiment 2).**

Item	Ileal tissue					Blood				
	8-OHdG pg/µL DNA	Methylation DNA, %	APE-1 ng/g	TDG ng/g	ANPG ng/g	8-OHdG pg/mL	Methylation DNA, %	APE-1 ng/mL	TDG ng/mL	ANPG ng/mL
Arg_90_*Infection (−)	23.40^d^	13.30^b^	1708.2^a^	983.2^a^	38.43^a^	4.136^b^	29.01	177.9^a^	93.44	4.937
Arg_90_*Infection (+)	79.03^ab^	17.69^a^	1000.9^b^	638.2^c^	20.34^b^	6.773^a^	66.25	148.7^b^	91.89	4.416
Arg_100_*Infection (−)	25.92^d^	13.44^b^	1494.4^a^	840.0^ab^	35.17^a^	5.369^ab^	29.02	159.7^ab^	91.28	5.053
Arg_100_*Infection (+)	88.68^a^	16.21^ab^	1008.9^b^	691.3^c^	21.44^b^	5.131^ab^	39.83	136.4^b^	85.37	4.577
Arg_110_*Infection (−)	38.15^c^	14.52^abc^	959.9^b^	666.9^bc^	19.34^b^	6.493^a^	29.22	150.0^b^	97.32	4.686
Arg_110_*Infection (+)	84.28^a^	13.01^bc^	951.8^b^	661.4^c^	25.55^b^	8.144^a^	74.74	159.6^ab^	94.07	3.999
SEM	4.152	0.383	48.35	22.98	1.377	0.243	2.863	3.103	1.340	0.102
Arg level, %										
90	51.21^b^	15.50	1354.6^a^	810.7^a^	29.38^a^	5.454^b^	47.63^ab^	163.3	92.66	4.677
100	57.30^a^	14.82	1251.6^a^	765.6^a^	28.30^a^	5.250^b^	34.43^b^	148.1	88.32	4.815
110	61.22^a^	13.77	955.9^b^	664.2^b^	22.44^b^	7.319^a^	51.98^a^	154.8	95.70	4.343
*C. perfringens infection*^1^										
–	29.16^b^	13.75^b^	1387.5^a^	830.0^a^	30.98^a^	5.333^b^	29.08^b^	162.5^a^	94.01	4.892^a^
+	84.00^a^	15.64^a^	987.2^b^	663.6^b^	22.44^b^	6.683^a^	60.27^a^	148.3^b^	90.44	4.331^b^
*P* value										
Arg	0.001	0.079	0.001	0.001	0.006	0.001	0.001	0.071	0.079	0.116
*Infection*	0.001	0.004	0.001	0.001	0.001	0.001	0.001	0.010	0.178	0.004
Arg × *infection*	0.001	0.001	0.001	0.001	0.001	0.003	0.001	0.009	0.789	0.889

Diets contained three levels of Arg relative to Lys (90, 100 and 110%; Arg_90_, Arg_100_ and Arg_110_, respectively) and a high level of Met (45% dietary Lys).

^1^At 34, 36 and 37 days of age, birds were infected with 1 mL (*per os* directly into the crop) of a culture medium of *C. perfringens* type A strain 56 containing approximately 10^8^ CFU/mL of bacteria. ^a,b,c^Means within a row with different superscripts differ significantly (*P* < 0.05).

Similarly to Experiment 1, *C. perfringens* infection increased the levels of MDA (*P* = 0.007) and ALB, and GPx activity (both *P* < 0.001), and decreased the levels of TG (*P* = 0.009) and UA, and ALT activity (both *P* < 0.001). In comparison with uninfected turkeys, infected birds were characterized by lower TP levels (*P* = 0.003), higher levels of TC (*P* < 0.001) and GLU (*P* = 0.006), and higher activity of CK (*P* < 0.001), AST (*P* = 0.003) and SOD (*P* < 0.001) (Table 8).

**Table 8:** **Metabolic markers in the blood of 42-day-old turkeys fed diets with different arginine (Arg) to lysine (Lys) ratios and a high methionine (Met) level, challenged with*Clostridium perfringens*—(Experiment 2).**

Item	CK U/L	ALT U/L	AST U/L	SOD U/gHb	GPx U/gHb	TP g/L	ALB g/L	UA mmol/L	UREA mmol/L	TC mmol/L	TG mmol/L	MDA µmol/L	GLU mmol/L
Arg_90_*Infection (−)	0.249^d^	13.13^a^	239.0^c^	807.2^c^	14.65^d^	44.59^a^	20.51^b^	0.412	1.319^ab^	3.727^ab^	0.621	1.718^b^	6.95^c^
Arg_90_*Infection (+)	0.365^b^	11.26^ab^	275.4^ab^	2234.1^a^	20.80^c^	36.05^b^	18.97^bc^	0.280	1.477^a^	3.498^b^	0.512	1.54^bc^	9.43^b^
Arg_100_*Infection (−)	0.187^e^	15.31^a^	247.7^bc^	1139.4^c^	14.44^d^	38.55^b^	19.99^b^	0.391	1.502^a^	2.945^c^	0.715	1.279^c^	10.25^b^
Arg_100_*Infection (+)	0.168^e^	6.64^c^	303.7^a^	2216.3^ab^	47.94^b^	38.1^b^	23.60^a^	0.347	1.146^ab^	4.063^a^	0.646	2.205^a^	10.32^b^
Arg_110_*Infection (−)	0.236 ^cd^	13.64^a^	289.3^a^	2126.6^ab^	12.92^d^	37.4^b^	18.39^c^	0.365	0.738^b^	3.358^bc^	0.396	1.830^b^	12.02^a^
Arg_110_*Infection (+)	0.434^a^	7.43^c^	255.0^bc^	1705.9^b^	55.60^a^	35.75^b^	22.58^a^	0.317	1.120^ab^	4.243^a^	0.336	2.119^a^	12.10^a^
SEM	0.014	0.678	4.438	93.23	2.576	0.676^b^	0.305	0.011	0.067	0.079	0.024	0.074	0.290
Arg level, %													
90	0.307^b^	12.20	257.2^b^	15207^c^	17.72^b^	40.32^a^	19.74^b^	0.346	1.398^a^	3.613	0.566^b^	1.629	8.19^c^
100	0.177^c^	10.98	275.7^a^	1677.8^b^	31.19^a^	38.33^ab^	21.80^a^	0.369	1.324^a^	3.504	0.681^a^	1.742	10.28^b^
110	0.335^a^	10.53	272.2^ab^	1916.3^a^	34.26^a^	36.58^b^	20.48^ab^	0.341	0.929^b^	3.800	0.366^c^	1.974	12.06^a^
*C. perfringens infection*^1^													
–	0.224^b^	14.03^a^	258.7^b^	1357.8^b^	14.00^b^	40.18^a^	19.63^b^	0.389^a^	1.187	3.343^b^	0.577^a^	1.609^b^	9.74^b^
+	0.322^a^	8.45^b^	278.0^a^	2052.1^a^	41.45^a^	36.64^b^	21.72^a^	0.315^b^	1.248	3.935^a^	0.498^b^	1.955^a^	10.62^a^
*P* value													
Arg	0.001	0.408	0.041	0.008	0.001	0.030	0.001	0.466	0.004	0.064	0.001	0.073	0.001
*Infection*	0.001	0.001	0.003	0.001	0.001	0.003	0.001	0.001	0.605	0.001	0.009	0.007	0.006
Arg × *infection*	0.001	0.034	0.001	0.001	0.001	0.009	0.001	0.124	0.040	0.001	0.755	0.003	0.002

Diets contained three levels of Arg relative to Lys (90, 100 and 110%; Arg_90_, Arg_100_ and Arg_110_, respectively) and a high level of Met (45% dietary Lys).

^1^At 34, 36 and 37 days of age, birds were infected with 1 mL (*per os* directly into the crop) of a culture medium of *C. perfringens* type A strain 56 containing approximately 10^8^ CFU/mL of bacteria. ^a,b,c^Means within a row with different superscripts differ significantly (*P* < 0.05).

#### Effects of dietary ratios of Arg to Lys

An increase in the dietary Arg:Lys ratio to 110% contributed to an increase in the BW of turkeys at 6 weeks of age (2.29 vs. 2.434 vs. 2.564 kg, *P* = 0.014). Different dietary inclusion levels of Arg, relative to Lys, had no influence on the expression of genes encoding intestinal barrier integrity in turkeys (Table 6). Similarly to Experiment 1, turkeys fed Arg_100_ and Arg_110_ diets had higher 8-OHdG levels in the wall of the small intestine. Turkeys receiving Arg_110_ diets were characterized by the highest 8-OHdG levels and the highest percentage of methylated DNA (both *P* < 0.001) in the blood. An increase in the Arg content to 110% Lys decreased the activity of APE-1, TDG and ANPG (*P* < 0.001) in the ileum. An Arg × infection interaction was noted for DNA methylation in the wall of the ileum (*P* < 0.001): *C. perfringens* infection increased DNA methylation at the lowest dietary Arg content (Arg_90_), but not at the medium or highest Arg content (Arg_100_ and Arg_110_, respectively). Similarly to Experiment 1, an Arg × infection interaction was observed for 8-OHdG levels in the blood (*P* = 0.003): *C. perfringens* infection increased 8-OHdG levels at the lowest dietary Arg content (Arg_90_) and both Met concentrations (30% or 45% Lys), but not at the medium or highest Arg content (Arg_100_ and Arg_110_, respectively). An Arg × infection interaction was also found for the activity of APE-1, TDG and ANPG in the wall of the ileum (*P* < 0.001): *C. perfringens* infection decreased the activity of the above enzymes at the lowest and medium dietary Arg content (Arg_90_ and Arg_100_, respectively) and Met concentration of 45% Lys, but not at the highest Arg content (Arg_110_). The following Arg × infection interaction was noted for APE-1 activity in the blood of turkeys (*P* = 0.009): *C. perfringens* infection decreased APE-1 activity at the lowest dietary Arg content (Arg_90_) and Met concentration of 45% Lys, but not at the medium or highest Arg content (Arg_100_ and Arg_110_, respectively) (Table 7).

Similarly to Experiment 1, an increase in the Arg content to 110% Lys increased SOD activity in the blood of turkeys (*P* = 0.008). Turkeys fed Arg_90_ diets had lower GPx activity and ALB levels (both *P* < 0.001) in the blood. An increase in the dietary inclusion rate of Arg (110% Lys) led to an increase in GLU levels and CK activity (both *P* < 0.001), and a decrease in UREA levels (*P* = 0.004) in the blood. An Arg × infection interaction was observed for the levels of ALB, TC (both *P* < 0.001) and MDA (*P* = 0.003) in the blood of turkeys: *C. perfringens* infection increased the levels of ALB, TC and MDA at the medium and highest Arg content (Arg_100_ and Arg_110_, respectively), but not at the lowest Arg content (Arg_90_). An Arg × infection interaction was also found for CK activity and UREA levels in the blood plasma of turkeys (*P* < 0.001, *P* = 0.04, respectively): *C. perfringens* infection increased CK activity and UREA levels at the lowest and highest Arg content (Arg_90_ and Arg_110_, respectively), but not at the medium Arg content (Arg_100_). The following Arg × infection interaction was noted for the activity of AST and SOD in the blood of turkeys (both *P* < 0.001): *C. perfringens* infection increased the activity of the above enzymes at the lowest and medium Arg content (Arg_90_ and Arg_100_, respectively), but not at the highest Arg content (Arg_110_). The following Arg × infection interaction was observed for ALT activity in the blood plasma of turkeys: *C. perfringens* infection decreased ALT activity at the medium and highest Arg content (Arg_100_ and Arg_110_, respectively), but not at the lowest Arg content (Arg_90_). The following Arg × infection interaction was found for the plasma levels of TP: *C. perfringens* infection decreased TP levels at the lowest Arg content (Arg_90_), but not at the medium or highest Arg content (Arg_100_ and Arg_110_, respectively). The following Arg × infection interaction was noted for the plasma levels of GLU: *C. perfringens* infection increased GLU levels at the lowest Arg content (Arg_90_), but not at the medium or highest Arg content (Arg_100_ and Arg_110_, respectively) (Table 8).

## Discussion

### Effects of infection

**Figure 1:**
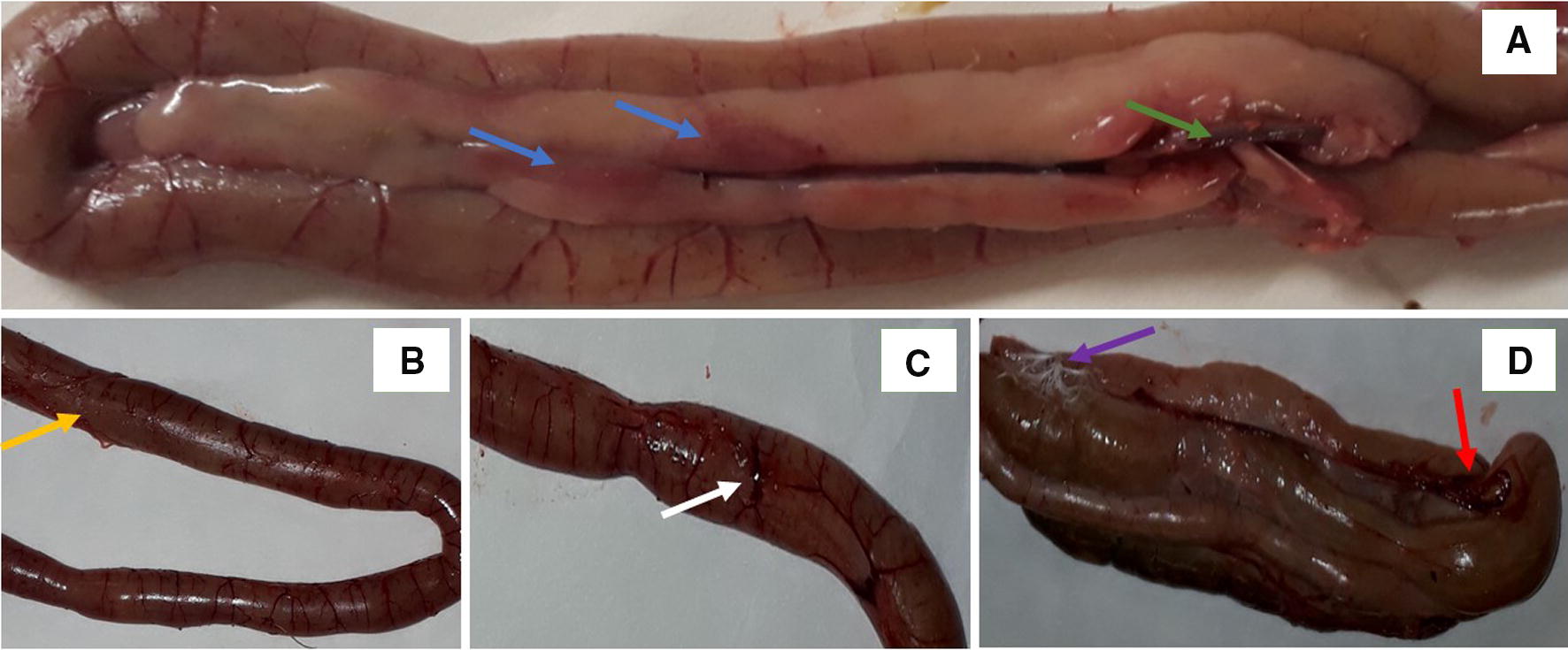
**Representative photographs illustrating changes in different intestinal segments of turkeys challenged orally with*C. perfringens***. The typical responses of the host included: **A** multifocal, minimal to mild hyperemia in the duodenum (blue arrow), and advanced necrosis in connective tissue (green arrow) in the duodenum; **B** lumpy coating reminiscent of a pseudomembrane (yellow arrow) in the jejunum; **C** manifested swollen vessels on the jejunum surface (white arrow); **D** multifocal hyperemia and hemorrhages (red arrow) and fibrinous coating on the surface of the caeca (purple arrow).

Intestinal barrier integrity plays a key role in protecting birds, in particular turkeys, against pathogenic microflora. Tight junction proteins (TJP) are the key molecules maintaining epithelial cell integrity. They enable the free passage of ions inside cells and prevent pathogens and their toxins from entering cells. sIgA and mucus flow are the main defenders of the intestinal mucosal barrier [26]. Claudin and occludin are integral membrane proteins in tight junctions [27] which form a tight barrier around cells and act as a physical barrier against the free flow of dissolved substances across intercellular spaces. Bacteria, including *C. perfringens,* secrete toxins and can compromise the tight junction barrier in the intestines, which leads to malabsorption. Therefore, the modulation of bacterial-mucosal cell interactions is of key importance in maintaining gut functional status. In the present report, we observed moderate necrotic lesions in different segments of the gut after *C. perfringens* administration (Figure 1). This finding confirmed that necrotic model consisting in *C. perfringens* administration in three periods preceded by coccidia challenge was successful at inducing gut inflammation, without causing bird death. Thus, it provides a good model for studying the specific interactions between diet and host response under challenge conditions. In the present study, the expression of occludin, ZO-1, GLP2, OGG1 and TFF2 genes increased in the wall of the small intestine, which indicates that *C. perfringens* infection in turkeys compromised intestinal barrier integrity. The expression of the above genes increased in birds fed diets with both low and high Met content, equivalent to 30% and 45% of Lys content, respectively. A review study by Awad et al. [28] indicates that *C. perfringens* endotoxin (CPE) increases intercellular permeability and disrupts intestinal mucosal barrier function in infected chickens. Cytotoxic CPE binds to claudin receptors and claudin-3 and claudin-4 cell monolayers (Madin-Darby Canine Kidney—MDCK) [29]. It has also been found that claudin proteins play an important role in the regulation of cell signaling. Claudin-1, -3 and -5, CLDN-16, ZO-1 and ZO-2 were expressed in the intestinal epithelium of chickens [30]. Occludin is often transported from tight junctions to cytoplasmic vesicles during the loss of intestinal barrier function [31], which can be stimulated by oxidative stress and inflammation [32]. Cani et al. [33] demonstrated that occludin expression was negatively correlated with the translocation of fluorescein isothiocyanate-dextran from the digestive system to the blood, which indicates that it plays a role in the maintenance of intestinal barrier integrity. Furuse et al. [27] also found that ZO-1 is directly associated with occludin. According to Saitoh et al. [34], CPE can bind to specific claudins to mediate the degradation of TJP and increase permeability across epithelial cells. Necrotic enteritis evokes oxidation processes, and it could be implicated in intestinal malabsorption. In this study, elevated MDA levels and decreased GPx activity in the blood suggest that *C. perfringens* also induced lipid oxidation in turkeys. Increased MDA concentration in the blood and other tissues, and decreased activity of antioxidant enzymes are indicative of lipid oxidation and impaired antioxidant defense mechanisms [11, 21]. According to Jankowski et al. [21], a higher inclusion rate of Met in turkey diets did not inhibit lipid oxidation during hemorrhagic enteritis. The compromised function of antioxidant defense mechanisms not only leads to lipid oxidation, but also to protein and DNA oxidation. In the current study, *C. perfringens* infection in turkeys also contributed to oxidative damage and epigenetic alterations leading to DNA damage, which were manifested by an increase in the levels of 8-OHdG in the wall of the small intestine and in the blood, and an increase in the percentage of methylated DNA in the blood. 8-OHdG, an oxidized guanine base adduct, is a major product of the oxidation of DNA bases by reactive oxygen species. Modified guanine can form stable base pairs with cytosine, which leads to its enzymatic methylation. The binding of methyl-binding proteins (MBP) is weakened when 8-OHdG is adjacent to 5-methylcytosine. Frequent conversion of 5-methylcytosine to 5-hydroxymethylcytosine also changes binding affinity for MBP, which leads to epigenetic modifications [35, 36].

This study demonstrated that the high Met level induced DNA methylation in the wall of the ileum, which was not observed in turkeys fed diets with low Met content. According to Young and Shalchi [37], Met is a precursor of S-adenosylmethionine, the main donor of methyl groups in a cell, which is responsible for DNA methylation, and DNA methylation protects microorganisms against the damaging effects of endonucleases and promotes the integrity of genomic DNA which is required for microbial proliferation. DNA methylation is an important physiological process, and its disruptions can lead to the methylation of genes encoding key proteins, such as antioxidant enzymes and DNA repair enzymes [38]. In this study, the activity of DNA repair enzymes (APE-1, TDG, ANPG) decreased in the wall of the small intestine and in the blood of turkeys infected with *C. perfringens*. In apurinic/apyrimidinic sites, APE1 is the main DNA repair enzyme in base excision repair (BER) and nucleotide excision repair (NER) pathways. In these sites, repair processes are initiated by DNA glycosylase enzymes which recognize modified bases, including OGG1 which recognizes 8-OHdG, ANPG which recognizes εA and εG, and TDG which recognizes εC. These enzymes remove the damaged base from the DNA strand at the site of damage [39]. In this experiment, TG levels in the blood decreased in infected turkeys fed diets with a 30% and 45% Met:Lys ratio. In the experiment performed by Abudabos et al. [40], *C. perfringens* infection in broilers did not affect GLU, TC or TG levels. According to Amad et al. [41], the infection did not lead to changes in the blood biochemistry of chickens. In our study, *C. perfringens* infection in turkeys administered diets with high and low Met content decreased the activity of liver enzyme ALT and UA. Numerous authors have demonstrated that viral and bacterial infections provoke hypertransaminasemia, but AST and ALT levels are quickly normalized or even decreased [42]. In infected turkeys, high-Met diets also increased TC and GLU concentrations, increased the activity of AST, SOD and CK, and decreased TP levels. These observations could be attributed to the effects of Met, rather than the infection because according to some authors, high dietary levels of Lys and Met can induce hypercholesterolemia in birds [43]. Previous studies of turkeys [21] and chickens [37] suggest that an increase in the dietary inclusion levels of Met can promote bacterial proliferation. Methionine could be a donor of methyl groups to the 5′-terminus of viral or bacterial mRNA, which promotes the rapid proliferation of pathogens [44]. Increased proliferation of *C. perfringens* under the influence of higher dietary Met content probably induced metabolic changes, including increased activity of AST, SOD and CK. The activity of these enzymes usually increases in response to induced oxidative stress [45]. Even though intestinal barrier integrity was compromised in young turkeys, *C. perfringens* infection had no effect on the growth performance of birds fed diets with high or low Met content. In many studies [46, 47], performance parameters decreased in *C. perfringens*–challenged chickens administered diets formulated according to NRC [23] recommendations.

### Effects of dietary ratios of Arg to Lys

Recent research has demonstrated that higher levels of amino acid influence GIT development and immune system in healthy and challenged broilers [14, 48]. According to the cited authors, higher intake of readily digestible amino acids can compensate for impaired intestinal absorption in challenged birds. Arginine, the key precursor of polyamine synthesis, can stimulate the proliferation, migration and apoptosis of intestinal cells, and promote mitotic processes in intestinal crypts and villi [49]. Tan et al. [14] found that the density of the intestinal mucosa increases linearly with a rise in dietary Arg levels, which could be attributed to the indirect effect of polyamines. However, it remains unknown whether Arg directly affects the replication of goblet cells or enterocytes.

Arginine is also a precursor of nitric oxide (NO), and it inhibits the replication of *Eimeria* parasites in the intestinal epithelium of chickens with coccidiosis [50]. In the present study, the administration of Arg_110_ diets containing 30% Met increased the expression of the OGG1 gene which conditions intestinal barrier integrity, but the above correlation was not observed in turkeys fed Arg_110_ diets containing 45% Met. OGG1 is the key repair enzyme which removes 8-OHdG from cellular DNA. The results of this study confirm that these amino acids regulate gene expression and the production of molecules that are essential for healthy gut function, including polyamines and NO [51, 52]. The role of Arg in mucin production and immune function has been extensively researched [14, 16] demonstrated that high levels of l-arginine limit disruptions in intestinal barrier integrity, which decreases the mRNA expression of claudin-1 and increases the mRNA expression of occludin.

The present findings indicate that Arg_90_ diets containing both 30% and 45% Met minimize DNA oxidation in the intestines. However, DNA oxidation and methylation was intensified in turkeys fed Arg_110_ diets, in particular diets containing 45% Met. At the same time, when the dietary inclusion level of Arg was increased to 110% of Lys content, oxidative and epigenetic changes were intensified in the intestines or in the blood, which was manifested by an increase in 8-OHdG levels and in the percentage of methylated DNA, and a decrease in the levels of DNA repair enzymes. According to Gao et al. [53], higher levels of Arg in poultry diets inhibit DNA oxidation and methylation. Therefore, the observed effect could be attributed to a higher dietary inclusion rate of Met. In the present study, Arg_110_ diets containing both 30% and 45% Met increased SOD activity in the blood of turkeys. Enhanced oxidation potentially mobilized the antioxidant enzyme to catalyze the dismutation of superoxide radicals in cells. Adverse changes were observed in selected blood biochemical parameters in turkeys fed Arg_110_ diets with a 30% Met:Lys ratio (higher TG and ALB levels) and a 45% Met:Lys ratio (higher CK activity and lower UREA concentration). These results are surprising since according to the literature, higher dietary supplementation levels of Arg decrease the plasma concentrations of GLU, TG and TCs and can promote the treatment of metabolic syndrome disorders [54]. In the current study, Arg_90_ diets with a 30% Met:Lys ratio had a favorable impact on liver metabolism by decreasing AST and ALT activity and increasing TP levels in the blood of turkeys. However, these diets also increased TC levels. Arg_90_ diets with a 45% Met:Lys ratio also decreased AST activity and inhibited oxidative processes, as demonstrated by a decrease in SOD and GPx activity and albumin levels. A review study by Fouad et al. [55] revealed that adequate dietary levels of Arg minimize oxidative stress, improve lipid and protein metabolism, and boost immunity in birds. In this study, diets with graded levels of Arg and a 30% Met:Lys ratio had no effect on the growth performance of turkeys, whereas Arg_110_ diets with a 45% Met:Lys ratio improved their growth rate. Adedokun et al. [56] reported higher feed conversion in challenged broiler chickens fed diets with increased concentrations of Lys, Met, threonine, isoleucine, tryptophan and valine. Jahanian and Khalifeh-Gholi [57] found that an increase in the dietary inclusion rate of Met improved FCR when the Arg content of chicken diets was increased to 110% of the level recommended by the NRC [23].

In turkeys infected with *C. perfringens*, fed diets with high Lys content, Arg content should be decreased to 90% Lys and Met content should be increased to 45% Lys. The above dietary amino acid ratios contribute to suppressing oxidative processes and epigenetic alterations in important molecules in the wall of the ileum and in the blood, and maintaining intestinal barrier integrity; they also exert a beneficial influence on metabolic parameters. Even though the analyzed amino acid ratios interacted with the systems responsible for the maintenance of gut integrity in the host organism, this dietary intervention probably enabled birds to cope with NE.

## Data Availability

The datasets used and/or analyzed during the current study are available from the corresponding author on reasonable request.

## Abbreviations

8-OHdG: 8-hydroxydeoxyguanosine
ALP: albumin
ALT: alanine aminotransferase
APE-1: endonuclease 1
Arg: arginine
BWG: body weight gain
CK: creatine kinase
CLDN15: Claudin 15
GLP2: Glucagon-like peptide-2
GPx: glutathione peroxidase
Lys: lysine
Met: methionine
NE: necrotic enteritis
NSP: non-starch polysaccharides
OCLN: Occludin
SOD: superoxide dismutase
TC: total cholesterol
TFF2: Trefoil Factor 2
TG: triglycerides
TP: total protein
UA: uric acid
ZO-1: Zonula occludens-1
